# Strategies for malaria vaccination during the COVID-19 pandemic in African countries

**DOI:** 10.2471/BLT.21.287472

**Published:** 2022-10-01

**Authors:** Kapil Narain, Sudhan Rackimuthu, Faisal A Nawaz, Osaretin Christabel Okonji, Henry Ashworth, Stefan S Du Plessis, Jaffer Shah

**Affiliations:** a Nelson R Mandela School of Medicine, University of KwaZulu-Natal, 719 Umbilo Rd, Umbilo, Berea, 4001 South Africa.; b Father Muller Medical College, Mangalore, Karnataka, India.; c College of Medicine, Mohammed Bin Rashid University of Medicine and Health Sciences, Dubai, United Arab Emirates.; d School of Pharmacy, University of the Western Cape, Cape Town, South Africa.; e Harvard Medical School, Boston, United States of America (USA).; f Drexel University College of Medicine, Pennsylvania, USA.

Since October 2021, the World Health Organization (WHO) recommends the use of RTS,S/AS01 (RTS,S) malaria vaccine for children in areas of moderate to high transmission of *Plasmodium falciparum* in Africa.^1^ The vaccine can reduce the 241 million cases of malaria and 627 000 malaria deaths worldwide;^2^^,^^3^ it is much needed in the WHO African Region, which accounts for 228 million cases of malaria (95% of global cases) and about 96% of global malaria deaths.^2^ However, an effective vaccine roll-out in Africa can only be achieved when region-specific challenges can be overcome; intraregional inequality, health-care systems strengthening and lessons from community engagement in previous public health crises.

The coronavirus disease 2019 (COVID-19) pandemic has posed an overwhelming burden on the fragile health-care systems in Africa and has disrupted various health-care services, including malaria prevention and treatment programmes.^4^^,^^5^ Isolated vaccination efforts are not sufficient to contain any disease, especially malaria. RTS,S prevents about one third of severe malaria cases in children younger than five years after administration of all four doses.^6^ However, a recent study showed that when used in conjunction with antimalarial drugs, full administration had a combined protective efficacy of 62.8% (95% confidence interval, CI: 58.4–66.8) against clinical malaria and 72.9% (95% CI: 2.9–92.4) against death from malaria.^7^

With the scale-up of RTS,S expected next year, the widespread intraregional inequality in Africa must be acknowledged and addressed. Africa is the second most unequal continent and home to seven of the most unequal countries; it is also experiencing a rapid rise in extreme poverty.^8^ Such inequality poses barriers in the effective deployment of RTS,S. In malaria-endemic regions, communities with poor health resources and limited access to malaria treatment and prevention should be prioritized. 

WHO’s Expanded Programme on Immunization plans to deliver the vaccine to all affected countries and requires collaborative efforts via sufficient funding and resource allocation. Because funding for COVID-19 might be temporary, donations from philanthropists and residual funds from governments, nongovernmental organizations and the International Monetary Fund should be reinvested into the malaria response. Health systems strengthening to improve malaria response can be achieved via capacity-building for production, procurement and distribution of malaria vaccines, antimalarials and other nonpharmacological modalities such as insecticide-treated nets and investments in the health workforce.

Health systems will need to create a short-term action plan for timely vaccine delivery to all malaria-endemic countries and areas, based on malarial burden. Delivery can be done by involving local social support organizations that can provide rapid workforce aid across communities. Regular surveillance of the malaria response and deployment of individuals with strong administrative capabilities to put in place robust logistic measures will also be necessary. Long-term plans involve implementation of health policies that focus on addressing the scalable costs of continued accessibility to malaria prevention and treatment modalities alongside reverse logistics systems. Such systems divert unused vaccines to areas of high demand, therefore ensuring that supplies reach those who are in need and are not wasted. Doing so requires coordinated efforts between health system governance constituents such as governmental health departments, international health organizations, pharmaceutical industries and partner institutions.

Stakeholders should consider key lessons from the Malaria Vaccine Implementation Programme, a pilot study in Ghana, Kenya and Malawi between 2019 and 2021.^9^ A similar programme is needed across Africa to promote the malaria vaccine with the support of partner organizations, wherein caregivers and communities are informed about the benefits and risks of the vaccine, eligibility, four-dose vaccination schedule and the importance of continuing nonpharmaceutical control measures.

Successful COVID-19 vaccine roll-outs bear important lessons. Community engagement is essential. Information can be disseminated to the public through health talks, meetings with community leaders and use of mainstream and social media to actively combat vaccine misinformation.^10^ Governments need strong commitment to develop national vaccine strategic plans and initiate capacity-building. Further research is needed to evaluate the public’s willingness to accept the malaria vaccine and to identify and address behavioural and social barriers. All research should be led or undertaken in equal partnership with malaria-endemic countries.

## References

[1] WHO recommends ground-breaking malaria vaccine for children at risk. Geneva: World Health Organization; 2021. Available from: https://www.who.int/news/item/06-10-2021-who-recommends-groundbreaking-malaria-vaccine-for-children-at-risk [cited 2021 Oct 10].

[2] World malaria report 2021. Geneva: World Health Organization; 2021. Available from: https://www.who.int/teams/global-malaria-programme/reports/world-malaria-report-2021 [cited 2021 Oct 10].

[3] WHO Director-General's opening remarks on WHO recommendation for wider use of the RTS,S malaria vaccine. Geneva: World Health Organization; 2021. Available from: https://www.who.int/director-general/speeches/detail/who-director-general-s-opening-remarks-on-who-recommendation-for-wider-use-of-the-rts-s-malaria-vaccine [cited 2021 Oct 15].

[4] Teboh-Ewungkem MI, Ngwa GA. COVID-19 in malaria-endemic regions: potential consequences for malaria intervention coverage, morbidity, and mortality. Lancet Infect Dis. 2021 Jan;21(1):5–6. 10.1016/S1473-3099(20)30763-5 32971007 PMC7505551

[5] Seboka B, Hailegebreal S, Kabthymer RH, Ali H, Yehualashet DE, Desalegn A et al. Impact of the COVID-19 pandemic on malaria prevention in Africa: evidence from COVID-19 health services disruption survey. Int J Trop Dis Health. 2021;9(6):287.

[6] RTS,S Clinical Trials Partnership. Efficacy and safety of RTS,S/AS01 malaria vaccine with or without a booster dose in infants and children in Africa: final results of a phase 3, individually randomised, controlled trial. Lancet. 2015 Jul 4;386(9988):31–45. 10.1016/S0140-6736(15)60721-8 25913272 PMC5626001

[7] Chandramohan D, Zongo I, Sagara I, Cairns M, Yerbanga RS, Diarra M, et al. Seasonal malaria vaccination with or without seasonal malaria chemoprevention. N Engl J Med. 2021 Sep 9;385(11):1005–17. 10.1056/NEJMoa2026330 34432975

[8] Seery E, Okanda J, Lawson M. A tale of two continents: fighting inequality in Africa. Nairobi: Oxfam Policy & Practice; 2019. Available from: https://policy-practice.oxfam.org/resources/a-tale-of-two-continents-fighting-inequality-in-africa-620856/ [cited 2022 Aug 7].

[9] Gavi, Unitaid and the Global Fund Welcome WHO Recommendation for World's first malaria vaccine. Geneva: The Global Fund; 2021. Available from: https://www.theglobalfund.org/en/news/2021-10-06-gavi-unitaid-and-the-global-fund-welcome-who-recommendation-for-worlds-first-malaria-vaccine/ [cited 2021 Oct 6].

[10] Malaria: the malaria vaccine implementation programme (MVIP). Geneva: World Health Organization; 2020. Available from: https://www.who.int/news-room/q-a-detail/malaria-vaccine-implementation-programme [cited 2021 Oct 6].

